# Ultrasensitive Photochemical Immunosensor Based on Flowerlike SnO_2_/BiOI/Ag_2_S Composites for Detection of Procalcitonin

**DOI:** 10.3390/bios11110421

**Published:** 2021-10-28

**Authors:** Nuo Zhang, Jinhui Feng, Guanhui Zhao, Xiaoyi Duan, Yaoguang Wang, Daopeng Zhang, Qin Wei

**Affiliations:** 1 School of Chemistry and Chemical Engineering, Shandong University of Technology, Zibo 255049, China; chm_zhangn@ujn.edu.cn (N.Z.); sennuo_1225@163.com (X.D.); 2 Key Laboratory of Interfacial Reaction & Sensing Analysis in Universities of Shandong, School of Chemistry and Chemical Engineering, University of Jinan, Jinan 250022, China; fengjinhui2011@163.com (J.F.); zhaoguanhui9001@163.com (G.Z.); sdjndxwq@163.com (Q.W.); 3 Shandong Provincial Key Laboratory of Molecular Engineering, School of Chemistry and Chemical Engineering, Qilu University of Technology (Shandong Academy of Sciences), Jinan 250353, China

**Keywords:** photoelectrochemical immunosensor, procalcitonin, SnO_2_, BiOI, Ag_2_S

## Abstract

Based on the necessity and urgency of detecting infectious disease marker procalcitonin (PCT), a novel unlabeled photoelectrochemical (PEC) immunosensor was prepared for the rapid and sensitive detection of PCT. Firstly, SnO_2_ porous nanoflowers with good photocatalytic performance were prepared by combining hydrothermal synthesis and calcining. BiOI nanoflowers were synthesized by facile ultrasonic mixed reaction. Ag_2_S quantum dots were deposited on SnO_2_/BiOI composites by in situ growth method. The SnO_2_/BiOI/Ag_2_S composites with excellent photoelectric properties were employed as substrate material, which could provide significantly enhanced and stable signal because of the energy level matching of SnO_2_, BiOI and Ag_2_S and the good light absorption performance. Accordingly, a PEC immunosensor based on SnO_2_/BiOI/Ag_2_S was constructed by using the layered modification method to achieve high sensitivity analysis of PCT. The linear dynamic range of the detection method was 0.50 pg·mL^−1^~100 ng·mL^−1^, and the detection limit was 0.14 pg·mL^−1^. In addition, the designed PEC immunosensor exhibited satisfactory sensitivity, selectivity, stability and repeatability, which opened up a new avenue for the analyzation of PCT and further provided guidance for antibiotic therapy.

## 1. Introduction

Due to its high accuracy for the diagnosis of bacterial infections, procalcitonin (PCT) is an FDA-approved blood infection marker for guiding antibiotic therapy [1,2]. In normal human serum, the level of PCT is less than 0.1 ng·mL^−1^ [3]. When the human body suffers from bacteria, fungi, or parasites and more severe sepsis or multiple organ failure, the level of PCT increases sharply. When PCT concentration in serum exceeds 0.5 ng·mL^−1^, the antibiotic therapy will be strongly recommended [4]. Research shows that elevated serum PCT is frequently seen in sepsis, severe trauma, heatstroke, necrotizing fasciitis, urinary tract infection, lower respiratory tract infections, neoplastic diseases and so on [5]. Some evidence also supports that PCT is also a new biomarker for the cardiologist [6]. In addition, PCT may be an indicator of disease severity in COVID-19 and may contribute to determine the severity of patients infected with SARS-CoV-2, thus playing an important role in COVID-19 antimicrobial stewardship [7,8]. Therefore, exploring an accurate, sensitive, simple and rapid determination method of PCT level in human blood serum is quite important for the early diagnosis of inflammation and other diseases, further guiding the antibiotic therapy.

Up to now, many detection methods based on immunoassay have been developed for PCT determination; for instance, enzyme-linked immunosorbent assay [9], chemiluminescence immunoassay [10,11], electrochemical immunoassay [12,13,14], microfluidics immunoassays [15], fluorescence immunoassay [16], lateral flow immunoassays [17], surface plasma resonance immunoassay [18], photoelectrochemical (PEC) immunosensor [19,20] and so on. Compared with other methods, PEC immunosensors have excellent features of high signal-to-noise ratio, simple operation, needlessness of expensive maintenance and easy miniaturization because of the dual advantages of optical and electrochemical methods. It is worth mentioning that label-free PEC immunosensors do not need complex labeling steps for the detected objects, which can save time, improve reproducibility and maintain the intrinsic characteristics of the objects.

As is known to all, the new photoelectrical sensing material plays an important role in the construction of the sensor [21,22]. Especially, metal oxide nanomaterials have good photoelectric and photocatalytic properties, which have been widely used in the field of PEC immunosensors. Among many oxide semiconductors, stannic oxide (SnO_2_) is one of the most representative semiconductor materials because of its unique catalytic and electrochemical properties, which has been widely applied in various domains including lithium-ion battery, solar cells, photocatalyst, photodetection and gas sensors [23,24,25,26,27]. A different morphology of SnO_2_ can be obtained by different synthesis methods and reaction conditions, such as nanoparticles, nanorods, nano-microspheres, nanoflowers and other microstructures. Flowerlike SnO_2_ is in favor of surface reaction and can enhance the sensing performances due to its structure configuration. Nonetheless, the wide band gap of SnO_2_ (Eg = 3.6 eV) leads to the poor light absorption properties and high electron-hole recombination. In order to improve the photocurrent conversion efficiency of SnO_2_, two basic approaches should be considered: one is to add the active sites of SnO_2_ by designing reasonable structure, and another is to build the heterostructure of SnO_2_ nanocomposites to promote charge separation effectively.

Environmentally friendly bismuth-based materials have attracted people’s great attention because of their special layered structure and excellent photocatalytic performance [28]. Among these materials, bismuth oxyiodide (BiOI) is a well-known layered p-type semiconductor (Eg = 1.7~1.9 eV), which has been widely used in the field of photocatalysis and pharmaceutical materials recently [29,30,31]. BiOI has strong absorption in the visible region and the alternates [Bi_2_O_2_]^2+^ layer and double I-layer can promote separation efficiency of photo-induced electrons and holes [32,33]. Through construction SnO_2_/BiOI heterojunction, excellent photocatalytic activity and efficient sunlight-harvesting nature can be realized. In addition, Ag_2_S nanoparticles with a narrower band gap (~1.0 eV) have also attracted great interests in the field of photocatalysis because of their excellent properties such as excellent stability, good electron transfer efficiency and prominent absorption coefficient, which are commonly employed in PEC fields [34]. With its good sensitization, Ag_2_S can be modified on the SnO_2_/BiOI electrode to further improve the photoelectric properties of the substrate material.

Herein, based on SnO_2_/BiOI/Ag_2_S nanocomposites, an ultrasensitive PEC immunosensor was developed for detection of PCT. Firstly, SnO_2_/BiOI/Ag_2_S composites were modified onto the ITO electrode, which can improve the sensitivity because of the high initial signal. SnO_2_ and BiOI can form N-P heterojunction with good photoactivity, and with the sensitization of Ag_2_S nanoparticles, the photocurrent response enhanced significantly. Secondly, anti-PCT and PCT were immobilized on the modified electrode by the reaction between carbonyl and amino groups and antibody–antigen specific recognition, respectively. Anti-PCT and PCT can generate a hydrophobic and insulating layer on the electrode surface, resulting in decrease of electron transfer and photocurrent intensity. According to the relationship between PCT concentration and photoelectric signal, the content of PCT in the unknown sample can be calculated.

## 2. Materials and Methods

### 2.1. Materials

PCT and anti-PCT were purchased from Linc-Bio Science Co., Ltd. (Shanghai, China). Indium tin oxide (ITO) glasses were obtained from Zhuhai Kaivo Electronic Components Co., Ltd. (Zhuhai, China) and cut into small pieces with a size of 2.5 × 1.0 cm^2^. ITO glasses were successively washed in acetone, ultrapure water, ethanol and ultrapure water under ultrasound before being used as working electrode. Other materials and instruments used are described in detail in the support materials.

### 2.2. Synthesis of SnO_2_

Flowerlike SnO_2_ was prepared by the reported method [35]. Briefly, 0.016 g of thioacetamide and 7 μL of anhydrous SnCl_4_ were added into a stainless-steel high-pressure reactor containing 10 mL of isopropanol. The mixture was ultrasonicated until completely dissolved. Then, the mixture was heated at 180 °C for 24 h and cooled naturally. Then, the products SnS_2_ were rinsed and dried and then calcined at 500 °C for 2 h in a muffle to obtain mesoporous SnO_2_.

### 2.3. Synthesis of BiOI

According to the literature [33], 1.0 mmol of KI was dissolved in 60 mL ultrapure water under stirring for 40 min. Then, 1.0 mmol of Bi(NO_3_)_3_·5H_2_O were added slowly drop by drop and treated with ultrasonic for at least 15 min. Subsequently, the mixtures were continuously stirred for about 3 h to obtain orange yellow powder. Finally, after washing and purification, the product BiOI was dried at 60 °C.

### 2.4. Fabrication of the PEC Immunosensor

**Figure 1:**
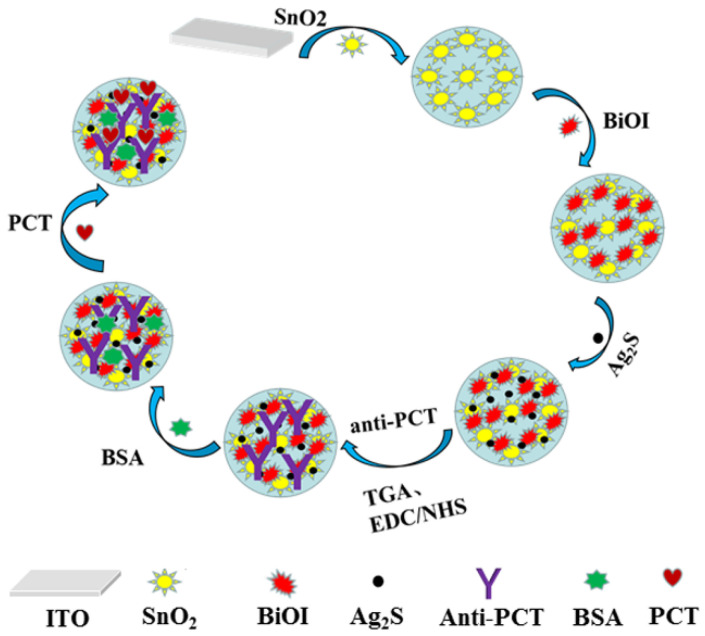
Illustration of the fabrication of immunosensor for detection of PCT.

Typically, 6 μL of SnO_2_ was dropped onto a piece of clean ITO and dried at room temperature. Then, 6 μL of BiOI aqueous solution with the concentration of 5 mg·mL^−1^ was coated on the surface of the ITO/SnO_2_. After being dried, the as-prepared ITO/SnO_2_/BiOI electrode was successively soaked 3 min for each step in 0.1 mol·L^−1^ AgNO_3_ solution in alcohol and 0.1 mol·L^−1^ Na_2_S in methanol aqueous solution (*V*/*V* = 1:1). Through this process, Ag_2_S was deposited on SnO_2_/BiOI composites by in situ growth method. Thereafter, 4 μL of thioglycolic acid (TGA) (3 mmol·L^−1^) was added on its surface, and then 6 μL of anti-PCT solution (1 μg·mL^−1^) was attached to the modified electrode. Subsequently, 4 μL of 0.1% BSA was used to block the unbound sites. At last, 6 μL of PCT solution with different concentrations was dropped onto the electrode and incubated with anti-PCT. The fabrication process of the immunosensor is shown in Figure 1.

### 2.5. PEC Detection of PCT

In this experiment, PEC detection was performed in PBS solution containing ascorbic acid (AA) and the light source used was LED lamp (working voltage was 36 V and operating current was 0~3.5 A). According to the relationship between the photocurrent intensity and the concentration of PCT, the quantitative PCT detection can be realized.

## 3. Results and Discussion

### 3.1. Characteristic Description of the Materials

**Figure 2:**
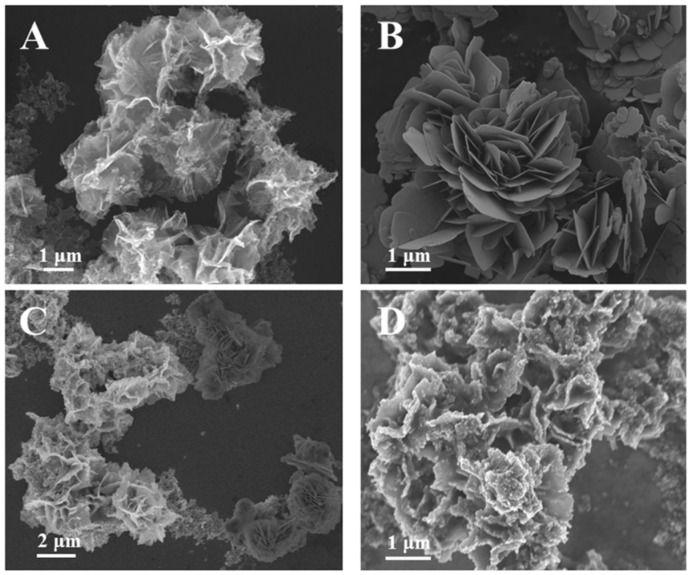
SEM images of SnO_2_ (**A**), BiOI (**B**), SnO_2_/BiOI (**C**), SnO_2_/BiOI/Ag_2_S (**D**).

The morphology of SnO_2_, BiOI, SnO_2_/BiOI and SnO_2_/BiOI/Ag_2_S was characterized by scanning electron microscopy (SEM). According to Figure 2A,B, it can be seen that both SnO_2_ and BiOI showed a flower structure composed of thin nanosheets with a size of about 2~3 μm, which could provide a large surface area for loading of more excellent performance of nanoparticles. Although both were flower-like structures, the pieces that make up the flower were not the same. In addition, in the atomic number contrast image, regions with large average atomic numbers were brighter than regions with small atomic numbers. According to the contrast of the sample with different atomic number, the morphology of the two samples can be clearly distinguished (Figure 2C). After SnO_2_/BiOI composites were successfully coated on the ITO electrode, Ag_2_S nanoparticles were uniformly deposited on its surface as sensitizer, which is shown in Figure 2D. We can see that the lamellar structure that made up the flower became thick due to the deposition of Ag_2_S. The element composition of the composites was measured with EDS spectrum, as shown in Figure S1. It can be seen that Sn, O, Bi, I, S and Ag elements were clearly observed, which further proved Ag_2_S was successfully fixed on the surface of SnO_2_/BiOI to obtain SnO_2_/BiOI/Ag_2_S composites. Furthermore, the EDS mapping images further confirmed the homogenous distribution.

**Figure 3:**
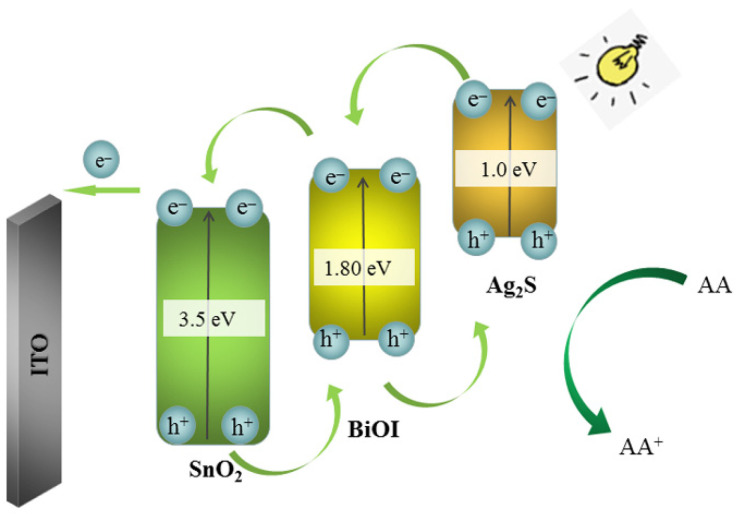
The possible photogenerated electron-hole transfer mechanism of the immunosensor.

Figure S2 shows the absorption of ultraviolet and visible light by the as-prepared SnO_2_, SnO_2_/BiOI and SnO_2_/BiOI/Ag_2_S composites. The absorption wavelength of SnO_2_ was less in the visible region, which was ascribed to its wide band gap. However, SnO_2_ could be coupled with BiOI, which resulted in the absorption peak shifting red, and the absorption peak became wider with strong absorption at 400~600 nm. Then, modification of Ag_2_S on the above electrode could improve the broad absorption in visible light obviously, further noticeably enhancing the photocurrent effect as expected. Observed from Figure S3, the photocurrent of SnO_2_/BiOI/Ag_2_S can be up to 93 μA, while SnO_2_, BiOI, SnO_2_/BiOI, SnO_2_/Ag_2_S, BiOI/Ag_2_S was 0.6 μA, 4 μA, 60 μA, 50 μA and 5 μA, respectively. Therefore, we selected SnO_2_/BiOI/Ag_2_S composites as the substrate material because of its excellent photoelectric property in this study. About the electron transfer mechanism, we suspected that SnO_2_, BiOI and Ag_2_S could form cascade band-edge levels because their energy bands were well matched (Figure 3), which promoted the separation of photogenic electrons and photogenic holes, resulting in ultrafast transfer of charge. In addition, the composite material has better light absorption in the visible region, resulting in a favorable utilization rate of solar energy.

### 3.2. Characterization of the PEC Immunosensor

**Figure 4:**
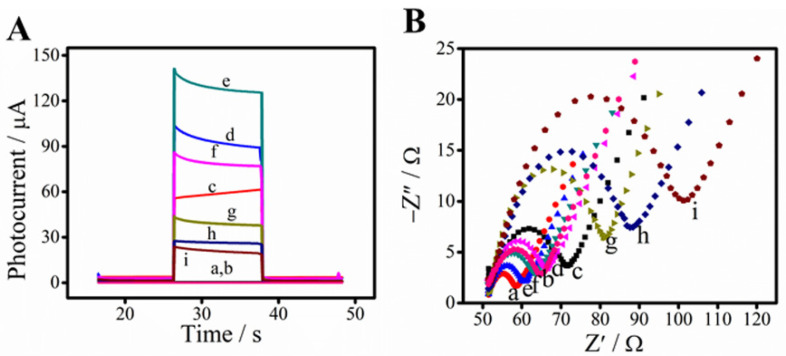
Photocurrent responses (**A**) and Nyquist plots of EIS (**B**). Different lines represent different materials to modify the electrode surface layer by layer: ITO electrode (a), after SnO_2_ modified (b), after BiOI modified (c), after Ag_2_S deposited (d), after carboxylation of TGA (e), after drops of EDC/NHS (f), after anti-PCT immobilization (g), after BSA blocking (h), after incubation with 0.1 ng·mL^−1^ of PCT (i).

The photocurrent variation trends of the modified electrodes are shown in Figure 4A, and the enlarged view of curve a and b is shown in Figure S4. After layer-by-layer modification, the photocurrent signal generated by the SnO_2_/BiOI/Ag_2_S modified electrode was significantly increased up to 104 μA (curve d), which was much higher than that of SnO_2_ and SnO_2_/BiOI (0.6 μA of SnO_2_ and 60 μA of SnO_2_/BiOI, curve b and c). This further confirmed the successful modification of Ag_2_S nanocomposites on the electrode surface. After the incubation of anti-PCT (curve g) and PCT (curve i) were modified onto the electrode in sequence, the photocurrents reduced from 45 μA to 20 μA. Part of the reason is that bioactive substances such as PCT, BSA and anti-PCT were insulating. Besides this, the increase in steric hindrance would block part of electron transfer to the photoactive substrate, leading to a gradual decline of photocurrent.

Electrochemical impedance spectroscopy (EIS) was applied to further analyze the interfacial properties of the proposed immunosensor, as shown in Figure 4B. The electron-transfer resistance (*R*_et_) value of ITO was small on account of the low electron transfer resistance (curve a). After the stepwise modification with SnO_2_ (curve b), BiOI (curve c), Ag_2_S (curve d), anti-PTC (curve g), BSA (curve h) and PCT (curve i), the *R*_et_ value changed significantly, which clearly verified the successful assembly process of the PEC immunosensor for PCT determination.

### 3.3. Optimal Conditions for PCT Detection

**Figure 5:**
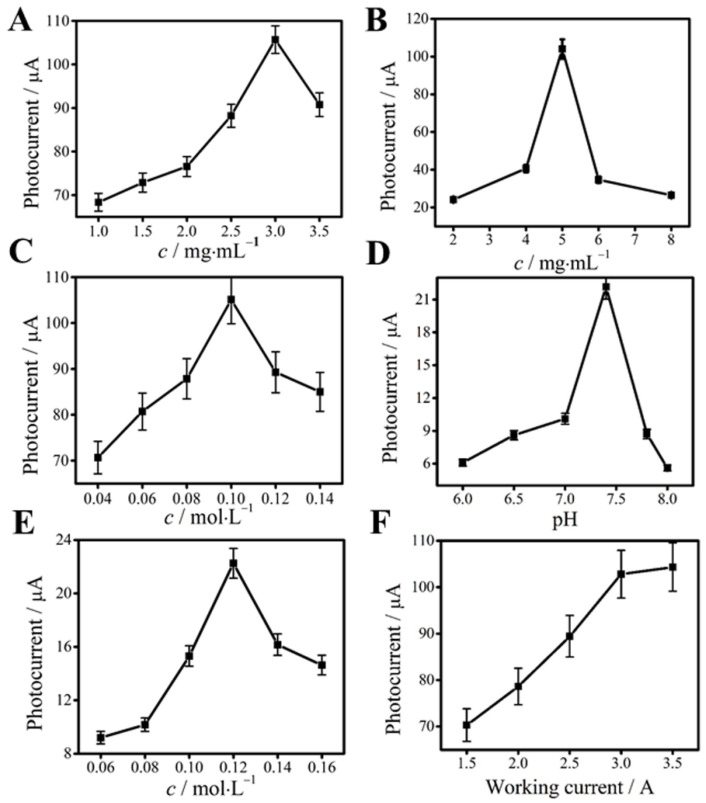
Optimal of concentration of SnO_2_ (**A**), BiOI (**B**), AgNO_3_ (**C**) and AA (**E**); pH value (**D**); intensity of the light source (**F**). Error bar = SD (*n* = 5).

The concentration of substrate material (SnO_2_, BiOI and AgNO_3_), the pH value, the concentration of AA and the light intensity are some essential experimental influence factors. From Figure 5A, we can see that when the concentration of SnO_2_ was too high, it probably blocked some of the electron transport. Hence, the SnO_2_ concentration of 3.0 mg·mL^−1^ was used in this study. Similarly, the optimal concentration of BiOI and AgNO_3_ was 5.0 mg·mL^−1^ and 0.10 mol·L^−1^ (Figure 5B,C). As shown in Figure 5D, pH 7.4 was selected as the best concentration. At this condition, the immobilized protein had the greatest activity. According to Figure 5E, when the concentration of AA was too low, fewer holes will be captured and there will be more electron-hole complexes in the system, so the photocurrent signal was small. However, when the concentration of AA in the solution was too high, the light intensity reaching the electrode surface decreased due to the absorption of the light intensity itself, making the excitation efficiency of Ag_2_S decrease, thus reducing the photocurrent signal of the whole system [36]. Therefore, 0.12 mol·L^−1^ of AA was chosen as the appropriate concentration in this system. Last but not the least, the photocurrent intensity increased with the increasing of the light intensity and reached a platform when the working current of LED lights was 3.0 A, which was selected as the optimal light intensity current in this experiment, as shown in Figure 5F.

### 3.4. PCT Detection

**Figure 6:**
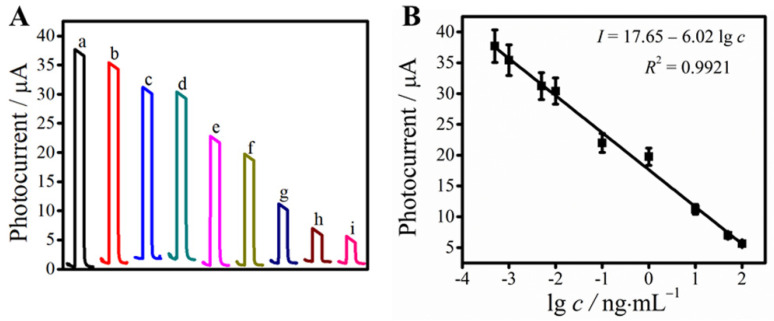
Photocurrent intensity of the immunosensor (**A**) and logarithmic calibration curve (**B**) of the PEC immunosensor at different PCT concentrations. The concentrations of PCT were 0.0005 (a), 0.001 (b), 0.005 (c), 0.01 (d), 0.1 (e), 1 (f), 10 (g), 50 (h), 100 (i) ng·mL^−1^; Error bars = SD (*n* = 5).

Under the above optimal experimental conditions, the detection of PCT was realized on the change of photocurrent intensity of the substrate materials, which attributed to the immunoreactions between PCT and anti-PCT and the changes of PCT concentration. According to Figure 6A, the photocurrent density and the logarithmic values of concentration of PCT had a good linear relationship with the range from 0.50 pg·mL^−1^ to 100 ng·mL^−1^. The linear equation was *I* = 17.65–6.02 lg *c*_PCT_ with a correlation coefficient of 0.9921 (Figure 6B). The limit of detection for PCT concentration was calculated to be 0.14 pg·mL^−1^ (S/N = 3). As shown in Table S1, the PEC immunosensor we constructed had a lower detection limit and a wider linear range than some other reported methods for the detection of PCT, for example, chemiluminescence immunoassay, electrochemical immunosensor, immunochromatographic assay, immunosorbent assay, time-resolved digital immunoassay and electrochemical paper-based analysis, but it was slightly inferior to electrochemiluminescence immunosensor. Compared with other existing PEC immunosensors, the as-prepared immunosensor still showed a satisfying performance in sensitivity and linear range.

### 3.5. Repeatability, Stability, Specificity and Application of the PEC Immunosensor

The repeatability, stability and specificity of biosensors are important indexes to measure the actual performance of biosensors.

The excellent repeatability is a prerequisite for accuracy. From Table S2, we can see that the relative standard deviation (RSD) of eleven parallel measurements was in the range of 2.2~8.4%, which demonstrated good repeatability.

The stability is critical to sensors. From Figure S5A, the photocurrent intensity was recorded with 16 light on/off cycles of incubation with 0.1 ng·mL^−1^ of PCT. There was no distinct change of photocurrent intensity value, indicating the prepared PEC immunosensor possessed the satisfactory stability for PCT detection.

To evaluate the specificity of the immunosensor for PCT, 100 ng·mL^−1^ of two typical interfering antigens, carcinoembryonic antigen (CEA) and prostatic specific antigen (PSA), were selected for interference test by mixing with 1.0 ng·mL^−1^ of PCT. According to Figure S5B, there was no obvious change of the photocurrent response in the case of the addition of CEA and PSA, which indicated that the satisfactory specificity.

To verify the viability of the PEC immunosensor for PCT detection in practical blood samples, the recovery experiments were carried out through adding different PCT standard solution to human serum samples. According to Table S2, we can see that the recoveries of the samples were in the range of 98.8~103%, and the RSD was in the range of 2.2~8.4%, which indicated the promising potential for the analyzation of PCT in the human blood serum samples.

## 4. Conclusions

In this study, based on the good photoelectric performance of SnO_2_/BiOI/Ag_2_S composites, a novel unlabeled PEC immunosensor was successfully constructed for PCT detection. The application of SnO_2_ in immunosensing analysis is still less, and SnO_2_ was a perfect substrate for PEC immunosensors due to its good stability on ITO electrode. Through construction SnO_2_/BiOI heterojunction, excellent photocatalytic activity and efficient sunlight-harvesting nature can be realized, and flower-like SnO_2_ and BiOI can provide large surface area for the loading of Ag_2_S and further sensitize to the photocurrent response. Under the optimal experimental detection conditions, the proposed immunosensor had a detection limit of 0.14 pg·mL^−1^ for PCT detection, and the linear range was 0.50 pg·mL^−1^~100 ng·mL^−1^, which showed a satisfying performance compared with some other reported methods. It is worth mentioning that SnO_2_/BiOI/Ag_2_S composites had some advantages such as convenient synthesis, low cost, excellent photoelectric performance and environmental friendliness, which have potential applications in the field of photoelectrocatalysis, photoelectric device research and so on.

## Data Availability

The data presented in this study are available on request from the corresponding author.

## Supplementary Materials

The following are available online at https://www.mdpi.com/article/10.3390/bios11110421/s1. Figure S1: The EDS mapping images of SnO_2_/BiOI/Ag_2_S composites; Figure S2: UV-Vis diffuse reflectance spectra of SnO_2_ (a), SnO_2_/BiOI (b) and SnO_2_/BiOI/Ag_2_S (c); Figure S3: Time-based photocurrent response curves of SnO_2_(a), BiOI(b), SnO_2_/BiOI(c), SnO_2_/Ag_2_S(d), BiOI/Ag_2_S(e) and SnO_2_/BiOI/Ag_2_S(f); Figure S4: Time-based photocurrent response curves of ITO electrode (a) and ITO/SnO_2_ (b); Figure S5: Stability curve of chronocurrent (A); Selectivity of the PEC immunosensor for detecting PCT (B): (a) Blank, (b) Blank + 100 ng·mL^−1^ CEA, (c) Blank + 100 ng·mL^−1^ PSA, (d) 1.0 ng·mL^−1^ PCT, (f) 1.0 ng·mL^−1^ PCT + 100 ng·mL^−1^ CEA, (g) 1.0 ng·mL^−1^ PCT + 100 ng·mL^−1^ PSA. The applied potential was 0 V. Error bars = SD (*n* = 5); Table S1: Comparison of the performance of the proposed PEC immunosensor for PCT detection and those of other reports; Table S2: The results of the PCT determination in human serum samples.
