# Supplementary material for: Ultrasensitive Photochemical Immunosensor Based on Flowerlike SnO2/BiOI/Ag2S Composites for Detection of Procalcitonin
Source: Biosensors (Basel). 2021 Oct 28;11(11):421. doi: 10.3390/bios11110421 (PMC8615900; doi:10.3390/bios11110421)
Supplement: Supplementary file 1 [file biosensors-11-00421-s001.zip › biosensors-1424617-SM-revised.pdf]

*Supplementary materials*

*Article*

# Ultrasensitive Photochemical Immunosensor Based on Flowerlike $SnO_2/BiOI/Ag_2S$ Composites for Detection of Procalcitonin

**Nuo Zhang [1,2], Jinhui Feng [2], Guanhui Zhao [2], Xiaoyi Duan [1], Yaoguang Wang [3,*], Daopeng Zhang [1,*] and Qin Wei [2]**

[1] School of Chemistry and Chemical Engineering, Shandong University of Technology, Zibo 255049, China; chm_zhangn@ujn.edu.cn (N.Z.); sennuo_1225@163.com (X.D.)
[2] Key Laboratory of Interfacial Reaction & Sensing Analysis in Universities of Shandong, School of Chemistry and Chemical Engineering, University of Jinan, Jinan 250022, China; fengjinhui2011@163.com (J.F.); zhaoguanhui9001@163.com (G.Z.); sdjndxwq@163.com (Q.W.)
[3] Shandong Provincial Key Laboratory of Molecular Engineering, School of Chemistry and Chemical Engineering, Qilu University of Technology (Shandong Academy of Sciences), Jinan 250353, China
* Correspondence: wangyaoguang@qlu.edu.cn (Y.W.); dpzhang@sdut.edu.cn (D.Z.);
Tel./Fax: +86-531-8276-7872

**Citation:** Zhang, N.; Feng, J.; Zhao, G.; Duan, X.; Wang, Y.; Zhang, D.; Wei, Q. Ultrasensitive Photochemical Immunosensor Based on Flowerlike $SnO_2/BiOI/Ag_2S$ Composites for Detection of Procalcitonin. *Biosensors* **2021**, *11*, 421. https://doi.org/10.3390/bios11110421

Received: 7 October 2021
Accepted: 25 October 2021
Published: 28 October 2021

**Publisher's Note:** MDPI stays neutral with regard to jurisdictional claims in published maps and institutional affiliations.

**Copyright:** © 2021 by the authors. Submitted for possible open access publication under the terms and conditions of the Creative Commons Attribution (CC BY) license (http://creativecommons.org/licenses/by/4.0/).

## 1. Materials and apparatus

Thioglycolic acid (TGA) was obtained from Macklin Reagent Co., Ltd. (Shanghai, China). 1-ethyl-3-(3-dimethylaminopropyl) carbodiimide hydrochloride (EDC) and N-hydroxysuccinimide (NHS) were obtained from Aladdin Reagent Database Inc. (Shanghai, China). Tin (IV) chloride pentahydrate ($SnCl_4$), thioacetamide, potassium iodide (KI), Bismuth nitrate pentahydrate ($Bi(NO_3)_3 \cdot 5H_2O$), sodium sulfide ($Na_2S$), silver nitrate ($AgNO_3$), ascorbic acid (AA), absolute ethanol, isopropyl alcohol and acetone were purchased from Sinopharm Chemical Reagent Co., Ltd. (Beijing, China). Phosphate buffered solution (PBS, 1/15 $mol \cdot L^{-1}$ $KH_2PO_4$ and 1/15 $mol \cdot L^{-1}$ $Na_2HPO_4$) containing AA was used as an electrolyte for the PEC measurements. All other chemicals in the experiment were analytical grade and were used as received without further purification.

## 2. Apparatus

Scanning electron microscope (SEM) images and energy dispersive spectroscopy (EDS) were obtained by using a field-emission SEM (Zeiss, Gemini 300, Germany). Electrochemical impedance spectroscopy (EIS) analysis was performed with an RST5200F electrochemical workstation (Zhengzhou Shiruisi Technology Co., Ltd, China) with a three-electrode system in a 5.0 $mmol \cdot L^{-1}$ $[Fe(CN)_6]^{3-/4-}$ solution containing 0.10 $mol \cdot L^{-1}$ KCl. UV-vis diffuse reflectance spectrum measurements were performed with a Shimadzu UV-3101PC spectrometer (Japan). All PEC experiments were measured on a CHI760E electrochemical workstation (Chenhua Instrument Shanghai Co., Ltd, China) by using a conventional three-electrode system comprising of a saturated calomel electrode as reference electrode, a platinum wire as a counter-electrode, and the as-prepared $SnO_2$/BiOI/$Ag_2S$ modified ITO electrode ($2.5 \times 1.0$ $cm^2$) as working electrode.

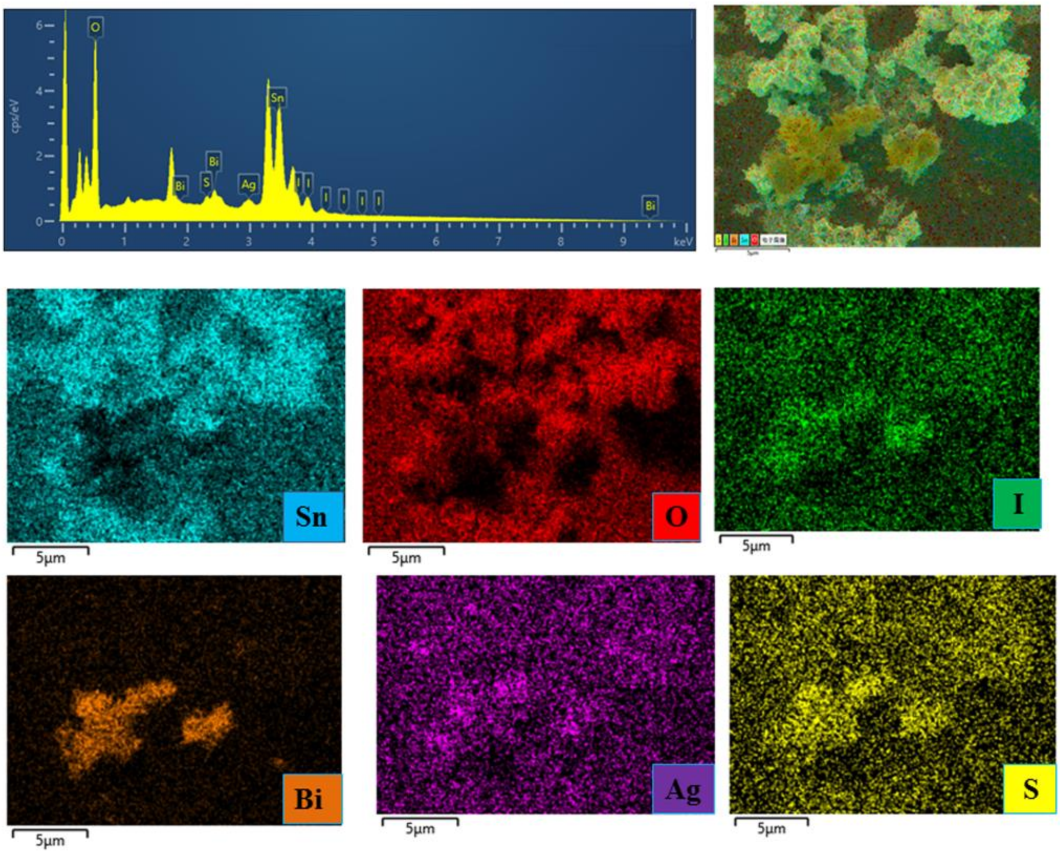


Figure S1 The EDS mapping images of $SnO_2$/BiOI/$Ag_2S$ composites.

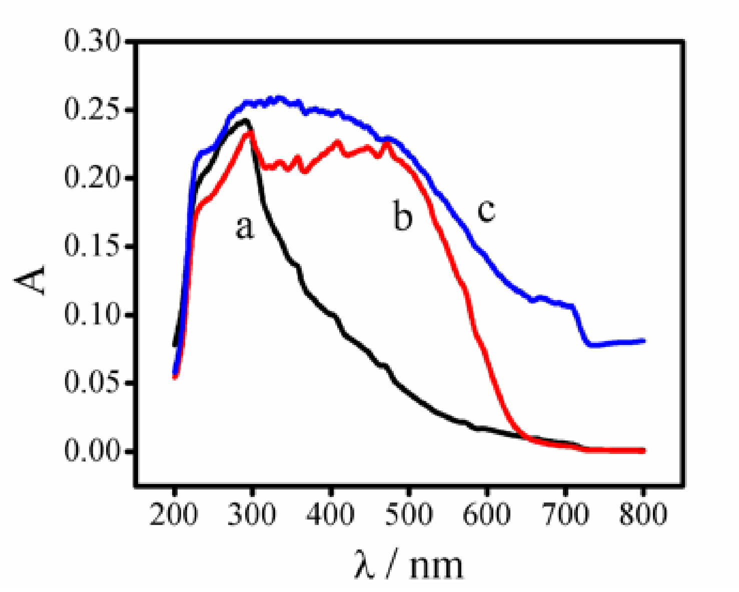


Figure S2 UV-Vis diffuse reflectance spectra of $SnO_2$ (a), $SnO_2$/BiOI (b) and $SnO_2$/BiOI/$Ag_2S$ (c).

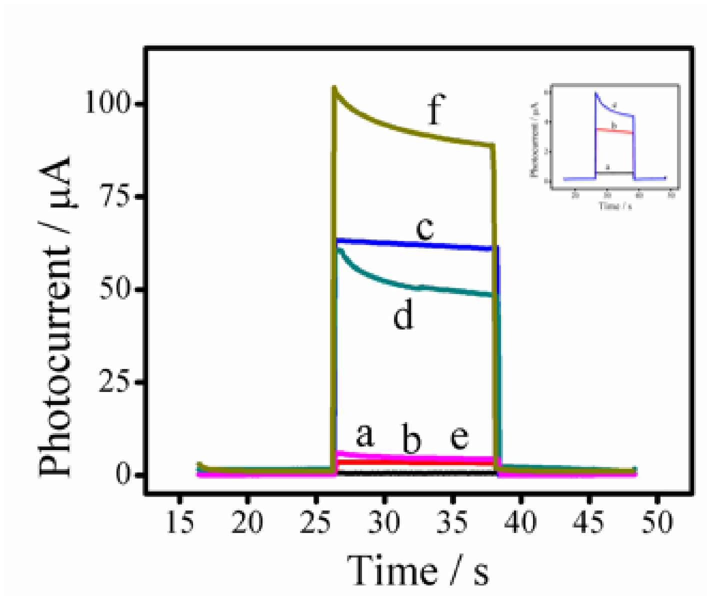


Figure S3 Time-based photocurrent response curves of $SnO_2$(a), BiOI(b), $SnO_2$/BiOI(c), $SnO_2/Ag_2S$(d), BiOI/$Ag_2S$(e) and $SnO_2$/BiOI/$Ag_2S$(f).

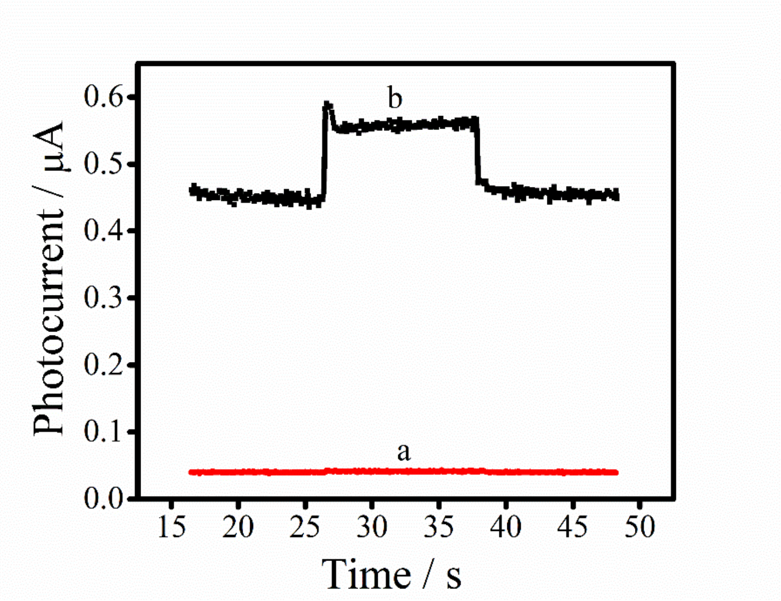


Figure S4 Time-based photocurrent response curves of ITO electrode (a) and ITO/$SnO_2$ (b)

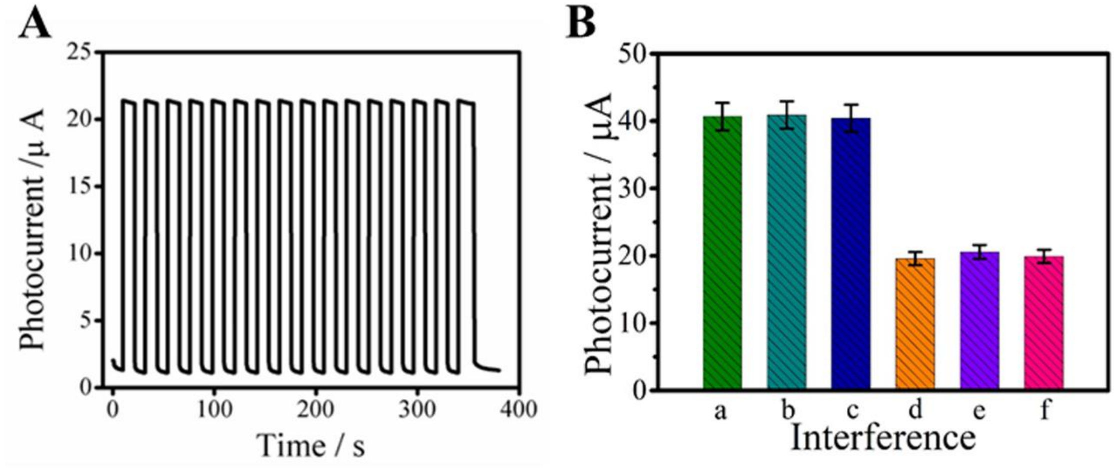


Figure 5. Stability curve of chronocurrent (A); Selectivity of the PEC immunosensor for detecting PCT (B) : (a) Blank , (b) Blank+100 ng·mL$^{-1}$ CEA, (c) Blank+100 ng·mL$^{-1}$ PSA, (d) 1.0 ng·mL$^{-1}$ PCT, (e) 1.0 ng·mL$^{-1}$ PCT+100 ng·mL$^{-1}$ CEA, (g) (f) 1.0 ng·mL$^{-1}$ PCT+100 ng·mL$^{-1}$ PSA. The applied potential was 0 V. Error bars = SD (*n* = 5).

Table S1 Comparison of the performance of the proposed PEC immunosensor for PCT detection and those of other reports.

| Detection method | Liner range ($pg{\cdot}mL^{-1}$) | Detection limit ($pg{\cdot}mL^{-1}$) | References |
|---|---|---|---|
| Chemiluminescence immunoassay | $44 - 1.0 \times 10^5$ | 44 | 1 |
| Eelectrochemical immunosensor | $1.0 - 5.0 \times 10^4$ | 0.36 | 2 |
| Ratiometric electrochemical immunosensor | $1.0 - 1.0 \times 10^5$ | 0.30 | 3 |
| Immunosorbent assay | $1.0 - 1.0 \times 10^5$ | 0.095 | 4 |
| Immunochromatographic assay | $62.5 - 4.0 \times 10^5$ | 62.5 | 5 |
| SERS-based immunochromatographic assay | $10 - 1.0 \times 10^7$ | 8.017 | 6 |
| Time-resolved digital immunoassay | $4.2 - 1.25 \times 10^4$ | 2.8 | 7 |
| Electrochemical paper-based analytical device | $0.50 - 2.5 \times 10^5$ | 0.27 | 8 |
| Lateral flow immunoassay | $12 - 1.0 \times 10^4$ | 31 | 9 |
| Capillary immunosensor | $0.10 - 1.0 \times 10^5$ | 0.01 | 10 |
| Electrochemiluminescence immunosensor | $0.10 - 1.0 \times 10^4$ | 0.0257 | 11 |
| Electrochemiluminescence immunosensor | $0.10 - 1.0 \times 10^5$ | 0.01258 | 12 |
| Electrochemiluminescence immunosensor | $0.010 - 1.0 \times 10^5$ | 0.00365 | 13 |
| Electrochemiluminescence immunosensor | 0.005 - 100 | 0.0021 | 14 |
| Electrochemiluminescence immunosensor | $0.10 - 5.0 \times 10^4$ | 0.054 | 15 |
| Electrochemiluminescence immunosensor | $0.05 - 1.0 \times 10^5$ | 0.017 | 16 |
| Photoelectrochemical immunosensor | $0.10 - 5.0 \times 10^4$ | 0.020 | 17 |
| Photoelectrochemical immunosensor | $0.10 - 1.0 \times 10^5$ | 0.030 | 18 |
| Photoelectrochemical immunosensor | $0.50 - 1.0 \times 10^5$ | 0.17 | 19 |
| Photoelectrochemical immunosensor | $1.0 - 1.0 \times 10^5$ | 0.42 | 20 |
| Photoelectrochemical immunosensor | $0.50 - 1.0 \times 10^5$ | 0.14 | This work |

Table S2. The results of the PCT determination in human serum samples

| Content in samples ($ng \cdot mL^{-1}$) | Added content ($ng \cdot mL^{-1}$) | Average content (*n*=11) ($ng \cdot mL^{-1}$) | RSD (*n*=11, %) | Recovery (%) |
|---|---|---|---|---|
| 0.0400 | 0.0100 | 0.0512 | 8.4 | 103% |
| | 1.00 | 1.04 | 7.6 | 100% |
| 0.800 | 1.00 | 1.79 | 4.7 | 98.8% |
| | 10.0 | 10.8 | 2.2 | 100 % |

## References：


[1] Enqi Huang, Dezhi Huang, Yu Wang, et al. Active droplet-array microfluidics-based chemiluminescence immunoassay for point-of-care detection of procalcitonin. Biosensors and Bioelectronics, 2022,195, 113684.

[2] Hui Ding, Lei Yang, Hongying Jia, et al. Label-free electrochemical immunosensor with palladium nanoparticles functionalized $MoS_2$/NiCo heterostructures for sensitive procalcitonin detection. Sensors and Actuators B: Chemical, 2020, 312, 127980.

[3] Juncong Miao, Kang Du, Xuan Li, et al. Ratiometric electrochemical immunosensor for the detection of procalcitonin based on the ratios of $SiO_2$-Fc-COOH-Au and UiO-66-TB complexes. Biosensors and Bioelectronics, 2021,171, 112713.

[4] Chang Yue Chiang, Tze Ta Huang, Chih Hui Wang, et al. Fiber optic nanogold-linked immunosorbent assay for rapid detection of procalcitonin at femtomolar concentration level. Biosensors and Bioelectronics, 2020, 151, 111871.

[5] Shu Zhou, Yuanli Peng, Jing Hu, et al. Quantum dot nanobead-based immunochromatographic assay for the quantitative detection of the procalcitonin antigen in serum samples. Microchemical Journal, 2020, 159, 105533.

[6] Ji Xia, Dan Lu, Yifan Liu, et al. Prediction of premature rupture of membranes via simultaneous detection of procalcitonin and interleukin-6 by a SERS-based immunochromatographic assay. New Journal of Chemistry, 2020, 44, 17099.

[7] Wenwen Jing, Yan Wang, Yunze Yang, et al. Time-resolved digital immunoassay for rapid and sensitive quantitation of procalcitonin with plasmonic imaging. ACS Nano, 2019, 13, 8609-8617.

[8] Suchanat Boonkaew, Ilhoon Jang, Eka Noviana, et al. Electrochemical paper-based analytical device for multiplexed, point-of-care detection of cardiovascular disease biomarkers. Sensors and Actuators B: Chemical, 2021, 330, 129336.

[9] Liang Huang, Yuxing Zhang,Tao Liao, et al. Compact magneto-fluorescent colloids by hierarchical assembly of dual-components in radial channels for sensitive point-of-care immunoassay. Small, 2021, 17, 2100862.

[10] Xuexue Xu, Xingda Song, Rongbin Nie, et al. Ultra-sensitive capillary immunosensor

combining porous-layer surface modification and biotin-streptavidin nano-complex signal amplification: Application for sensing of procalcitonin in serum. Talanta, 2019, 205, 120089.

[11] Cui Song, Xiaojian Li,Lihua Hu, et al. Quench-Type Electrochemiluminescence Immunosensor Based on Resonance Energy Transfer from Carbon Nanotubes and Au-Nanoparticles-Enhanced g-$C_3N_4$ to CuO@Polydopamine for Procalcitonin Detection. ACS Applied Materials & Interfaces, 2020, 12, 8006-8015.

[12] Lihua Hu, Cui Song,Tengfei Shi, et al. Dual-quenching electrochemiluminescence resonance energy transfer system from IRMOF-3 coreaction accelerator enriched nitrogen-doped GQDs to ZnO@Au for sensitive detection of procalcitonin. Sensors and Actuators B: Chemical, 2021, 346, 130495.

[13] Lu Zhao, Xianzhen Song, Xiang Ren, et al. Ultrasensitive near-infrared electrochemiluminescence biosensor derived from Eu-MOF with antenna effect and high efficiency catalysis of specific $CoS_2$ hollow triple shelled nanoboxes for procalcitonin. Biosensors and Bioelectronics, 2021, 191, 113409.

[14] Xiaojian Li, Yu Du, Peng Xu, et al. Signal-off electrochemiluminescence immunosensor based on Mn-Eumelanin coordination nanoparticles quenching PtCo-$CuFe_2O_4$-reduced graphene oxide enhanced luminol. Sensors and Actuators B: Chemical, 2020, 323, 128702.

[15] Lei Yang, Jingwei Xue, Yue Jia, et al. Construction of well-ordered electrochemiluminescence sensing interface using peptide-based specifific antibody immobilizer and N-(aminobutyl)-N-(ethylisoluminol) functionalized ferritin as signal indicator for procalcitonin analysis. Biosensors and Bioelectronics, 2019, 142, 111562.

[16] Xinrong Shao, Xianzhen Song, Xin Liu, et al. A dual signal-amplified electrochemiluminescence immunosensor based on core-shell $CeO_2$-Au@Pt nanosphere for procalcitonin detection. Microchimica Acta, 2021, 188, 344.

[17] Yanrong Qian, Jinhui Feng, Huan Wang, et al. Sandwich-type signal-off photoelectrochemical immunosensor based on dual suppression effect of PbS quantum dots/$Co_3O_4$ polyhedron as signal amplification for procalcitonin detection.

Sensors and Actuators B: Chemical, 2019, 300, 127001.

[18] Chunzhu Bao, Dawei Fan, Xin Liu, et al. A signal-offff type photoelectrochemical immunosensor for the ultrasensitive detection of procalcitonin: $Ru(bpy)_3{}^{2+}$ and $Bi_2S_3$ co-sensitized $ZnTiO_3/TiO_2$ polyhedra as matrix and dual inhibition by $SiO_2$/PDA-Au. Biosensors and Bioelectronics, 2019, 142, 111513.

[19] Mengdi Wang, Xuejing Liu, Hongying Jia, et al. A novel approach to photoelectrochemical immunoassay for procalcitonin on the basis of $SnS_2$/CdS. New Journal of Chemistry, 2020, 44, 15281.

[20] Xin Liu, Chunzhu Bao, Xinrong Shao, et al. A procalcitonin photoelectrochemical immunosensor: NCQDs and $Sb_2S_3$ co-sensitized hydrangea-shaped $WO_3$ as a matrix through a layer-by-layer assembly. New Journal of Chemistry, 2020, 44, 2452.
